# Role of glucagon-like peptide-1 receptor agonists in type 2 diabetes-associated atherosclerosis: from vascular mechanism to omics-based biomarkers and imaging

**DOI:** 10.3389/fcvm.2026.1813873

**Published:** 2026-05-14

**Authors:** Raghad Al-Taweel, Ahmed Malki

**Affiliations:** 1 Department of Biomedical Sciences, College of Health Sciences, QU Health, Qatar University, Doha, Qatar; 2 Biomedical Research Centre, Qatar University, Doha, Qatar

**Keywords:** atherosclerosis, biomarkers, cardiometabolic disease, endothelial dysfunction, GLP-1RA, macrovascular complications, oxidized LDL (oxLDL), type 2 diabetes

## Abstract

Atherosclerosis is a chronic metabolic disorder driven by endothelial dysfunction, inflammation, oxidative stress, and progressive plaque remodeling; processes amplified in type 2 diabetes. Glucagon-like peptide-1 receptor agonists (GLP-1RAs), originally developed for glycemic control, have emerged as modulators of vascular biology with potential anti-atherosclerotic effects. This review synthesizes evidence across key mechanistic domains, including endothelial dysfunction, inflammatory signaling, oxidative stress pathways, plaque biology, extracellular matrix remodeling, and immune-cell modulation. GLP-1RAs reduce circulating inflammatory mediators, suppress NLRP3 inflammasome activity, and lower oxidized LDL, thereby limiting the initiation of vascular injury. At the endothelial level, they enhance nitric oxide (NO) availability, decrease NOX-derived oxidative stress, and reduce adhesion molecule expression, collectively improving vascular function. Within plaques, GLP-1RA signaling alters macrophage behavior, promotes cholesterol efflux, and modulates metalloproteinase activity, suggesting potential effects on plaque composition and stability. Emerging biomarker platforms, including microRNA profiling and high-throughput proteomic and lipidomic signatures, together with advanced imaging approaches such as MRI-visible nano-GLP-1RA formulations, provide novel tools to monitor molecular and spatial drug effects *in vivo*. Collectively, these findings position GLP-1RAs as modulators of atherosclerotic disease beyond glycemic control, with integrated effects spanning systemic immunometabolism and plaque biology. By linking mechanistic insights with emerging imaging and multi-omics technologies, this review highlights a path toward biomarker-driven patient stratification and precision cardiovascular therapeutics, redefining how vascular risk is assessed and treated in metabolic disease.

## 1 Introduction

Atherosclerosis is a chronic metabolic and inflammatory disorder marked by endothelial dysfunction, lipid oxidation, immune cell recruitment, and plaque formation (1). Endothelial dysfunction is driven by oxidative stress, inflammation, and reduced nitric oxide (NO) bioavailability (2). Oxidized LDL promotes these processes through LOX-1—mediated ROS generation and downstream NF-κB activation, contributing to early plaque formation and progression (3). In parallel, matrix metalloproteinases and inflammatory cytokines regulate extracellular matrix remodeling and plaque progression (3–5).

**Table 1:** Summary of the effects of GLP-1 receptor agonists (GLP-1RA) on discussed biomarkers.

Category	Biomarker	Effect by GLP-1RA	Evidence type
Inflammatory markers	TNF-α, IL-1β, IL-6, IL-12, IL-10, and CRP	↓ Significant reduction	Clinical, Pre-Clinical (3–5, 7)
Endothelial activation markers	ICAM-1, VCAM-1, MCP-1, P-selectin, L-selectin	↓ Significant reduction	Clinical, Pre-Clinical (3, 7, 14)
Oxidative stress markers	ROS, NOX4	↓ Significant reduction	Clinical, & Pre-Clinical (3, 7)
Antioxidant markers	NO, SOD	↑ Significant increase	Pre-Clinical (3)
Matrix remodeling markers	MMP-9	↓ Significant reduction	Clinical (4)
Matrix remodeling endogenous inhibitors	TIMP-1	↑ Significant increase	Clinical (4)
Plasma biomarkers	OPG	↑Significant increase	Clinical (4)
Cardiovascular risk markers	NT-proBNP, ANGPT2, THBS2, MSR1, and TNC	↓ Significant reduction	Proteomic Cohort (26)

Biomarkers are grouped by biological function: inflammatory markers (TNF-α, IL-1β, IL-6, IL-12, IL-10, CRP), endothelial activation markers (ICAM-1, VCAM-1, MCP-1, P-selectin, L-selectin), oxidative stress markers (ROS, NOX4), antioxidant markers (NO, SOD), matrix remodeling markers (MMP-9), matrix remodeling endogenous inhibitors (TIMP-1), plasma biomarkers (OPG), and Cardiovascular risk markers (NT-proBNP, ANGPT2, THBS2, MSR1, and TNC). Effects of GLP-1RA are indicated as↓ a significant reduction or ↑ a significant increase.

Glucagon-like peptide-1 (GLP-1), an incretin hormone, regulates glucose metabolism and has emerging roles in vascular function and inflammation. GLP-1 receptor agonists (GLP-1RAs), including clinically approved agents such as semaglutide, liraglutide, and dulaglutide, are widely used for glycemic control in patients with type 2 diabetes and have demonstrated cardiovascular benefits (4, 6). Beyond metabolic regulation, accumulating evidence suggests that GLP-1RAs modulate inflammatory signaling, reduce oxidative stress, and influence vascular remodeling pathways implicated in atherosclerosis (4). Preclinical and clinical studies indicate that GLP-1RAs are associated with reduced vascular inflammation and may contribute to favorable plaque characteristics, particularly in T2D populations at high cardiovascular risk, suggesting potential vascular benefits beyond glycemic control (4, 7). This review synthesizes recent original studies examining the biochemical mechanisms and emerging detection technologies through which GLP-1RAs may influence atherosclerosis in diabetic patients. By integrating evidence from both clinical and preclinical studies, this review aims to provide a translational perspective on the potential role of GLP-1RAs in the prevention and management of atherosclerotic cardiovascular disease as a macrovascular complication of T2D. Existing reviews largely focus on cardiovascular outcome benefits without fully addressing whether GLP-1RAs exert direct plaque-level effects independent of systemic metabolic improvements. This review addresses this gap by integrating molecular, imaging, and omics-based evidence.

## 2 Mechanistic insights into GLP-1RA actions in atherosclerosis

GLP-1RA influences multiple stages of atherosclerosis, including inflammation, endothelial dysfunction, and lipid metabolism, through interconnected molecular pathways. Studies have shown that GLP-1RA affects the key phases by reducing its main cytokines and chemokines, such as TNFα, IL-6, IL-10, and IL-12, and MCP-1, leading to a reduced inflammatory state, improved endothelial function, and promoted cholesterol efflux. These effects have been consistently observed across commonly used GLP-1RAs, including semaglutide and liraglutide.

### 2.1 Anti-inflammatory signaling and antioxidant effect

Low-grade vascular inflammation is a central driver of atherogenesis, promoting monocyte recruitment, cytokine secretion, and plaque progression (8). Monocyte recruitment and differentiation into macrophages represent key early events in atherogenesis, driving foam-cell formation and sustaining vascular inflammation. Accumulating evidence indicates that GLP-1RAs attenuate these inflammatory pathways, thereby slowing plaque development. Studies have demonstrated that following GLP-1RA treatment, inflammatory cytokines, including TNFα, IL-1β, IL-6, IL-10, and IL-12, undergo significant modulation, generally reflecting an anti-inflammatory shift (3, 5). In a 2024 clinical study involving patients with diabetes and atherosclerosis, GLP-1RA therapy was shown to significantly reduce initiating biomarkers such as oxLDL, pro-inflammatory cytokines, interleukin-1β (Il-1β), and tumor necrosis factor α (TNFα) (5). oxLDL plays a pivotal role in the onset of atherosclerosis as it can be recognized by T lymphocytes within the vascular wall, which triggers an antigen-dependent immune response that contributes to endothelial injury and early plaque development (9, 10). IL-1β activation in the vessel wall is linked to stimulation of the nucleotide-binding oligomerization domain-like receptor, pyrin domain-containing 3 (NLRP3) inflammasome following cholesterol crystal accumulation, resulting in the conversion of pro-IL-1β into its active form and promoting lesion progression (11, 12). Similarly, TNF-α contributes to atherosclerotic plaque formation through mechanisms closely tied to oxidative stress, ultimately heightening cardiovascular risk (13). The observed reductions in oxLDL, TNFα, and IL-1β following dulaglutide or semaglutide therapy underscore the capacity of GLP-1RAs to suppress key inflammatory mediators involved in plaque initiation and progression, positioning these agents as important tools for mitigating cardiovascular risk (5). Moreover, another clinical study in 2023 have shown decreased plasma IL-6, TNF-α, IL-12, and IL-10 in comparison to diabetic patients not treated with GLP-1RA (7). Results have also shown that GLP-1RA therapy reduces mitochondrial ROS, indicating improved oxidative balance (7). The reduced ROS was also observed in (3) study in which it was associated with increased expression of NO, and superoxide dismutase (SOD), which are antioxidants against ROS.

### 2.2 Improvement of endothelial function and reduction of adhesion molecules

Endothelial activation is an early biochemical event in atherosclerosis, characterized by increased expression of adhesion molecules, including Intercellular Adhesion Molecule 1 (ICAM-1), vascular Cell Adhesion Molecule 1 (VCAM-1), Monocyte Chemoattractant Protein-1 (MCP-1), and L-selectin. These molecules facilitate leukocyte adhesion and trans endothelial migration, thereby accelerating plaque development. GLP-1RA treatment has been shown to exert a beneficial effect on the expression of adhesion molecules, which facilitate endothelial healing.

A recent mechanistic study using LDL receptor—deficient mice and ox-LDL—challenged human endothelial cells demonstrated that liraglutide, one of the most widely studied GLP-1RAs, directly improves endothelial function by suppressing oxidative and inflammatory pathways (3). In LDLR-KO mice, liraglutide enhanced acetylcholine-induced vasodilation, reduced aortic LOX-1 expression, and lowered circulating oxidative and inflammatory markers; in which these effects were fully abolished by co-administration of the GLP-1R antagonist exendin-9, confirming that these vascular benefits are GLP-1R-dependent. Parallel *in vitro* experiments showed that liraglutide mitigated oxLDL-induced endothelial injury by suppressing NOX4 and NF-κB signaling, and downregulating ICAM-1 and VCAM-1 expression (3). Importantly, another clinical study evaluated leukocyte-endothelial interaction and adhesion molecules in T2D patients using isolated polymorphonuclear leukocytes (PMNs), showing lower rolling flux and adhesion molecules, including ICAM-1, V-CAM, and P-selectin, along with increased rolling velocity, indicating less adhesion activity (7). A separate clinical study measuring circulating adhesion molecules by ELISA similarly reported significant reductions in ICAM-1, VCAM-1, and L-selectin following GLP-1RA therapy (14). Also, MCP-1 was measured and showed reduced levels following GLP-1RA therapy, indicating decreased monocyte recruitment to the vascular wall (14). These results indicate that following GLP-1RA treatment, leukocyte–endothelial interactions will be lowered, aligning with mechanistic data indicating enhanced nitric oxide (NO) bioavailability and decreased oxidative stress downstream of GLP-1R activation.

### 2.3 GLP-1RA modulation of promotion cholesterol efflux

Beyond inflammation, impaired cholesterol efflux from macrophage-derived foam cells, mediated by key transporters such as ABCA1 and ABCG1, contributes to intracellular lipid accumulation and promotes plaque progression and instability (15). Several studies indicate that GLP-1RAs can favorably modulate lipid metabolism at the plaque level.

A study in 2022 demonstrated that targeted GLP-1RA nanoparticles directly reduced vascular inflammation in ApoE⁻/⁻ mice, even at doses too low to affect glycemia or body weight (16). The nanoparticles were found to preferentially accumulate in plaque-resident cells, including VSMC-like cells, and targeted cells exhibited a lower inflammatory status, as shown by decreased *Tnf* and *Icam1* expression with a concomitant increase in *Arg1*, a marker of alternative macrophage activation. The engineered drug localized to lipid-rich plaque regions and CD11b⁺/CD11c⁺ inflammatory cells, effectively suppressing inflammatory responses and enhancing cholesterol efflux (16).

**Figure 1:**
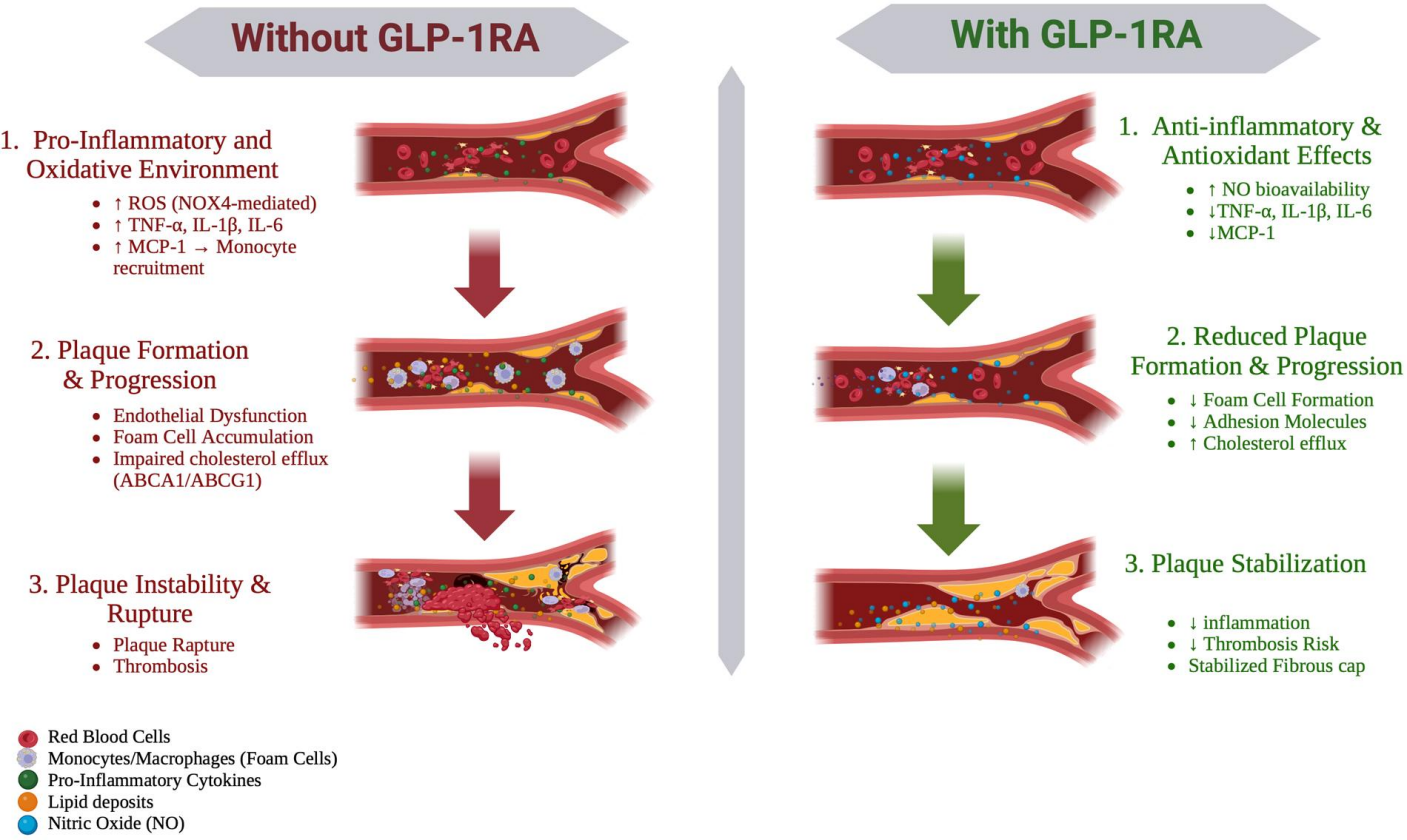
Schematic representation of the multi-level effects of GLP-1 receptor agonists (GLP-1RAs) on atherosclerosis progression. In the absence of GLP-1RA therapy, increased oxidative stress, inflammatory cytokine signaling, and MCP-1-mediated monocyte recruitment promote macrophage activation, foam-cell formation, and impaired cholesterol efflux, leading to plaque progression and instability. In contrast, GLP-1RA therapy reduces oxidative stress, suppresses inflammatory cytokines, enhances nitric oxide (NO) bioavailability, and improves cholesterol efflux, resulting in reduced plaque progression and enhanced plaque stability. Created with Biorender.com.

By promoting cholesterol efflux and reducing foam-cell content within plaques, GLP-1RA therapy may decrease lipid core size and necrotic burden, thereby stabilizing the fibrous cap, lowering the risk of plaque rupture, and potentially reducing acute coronary events. These preclinical findings align with clinical evidence from diabetic patients showing that GLP-1RA therapy lowers circulating MCP-1, a key chemokine that drives monocyte recruitment and promotes foam-cell formation (14). Together, these results suggest that GLP-1R activation remodels systemic and plaque-localized inflammatory and lipid-handling pathways in a manner that may directly contribute to plaque stabilization and reduced cardiovascular risk. A schematic overview summarizing the multi-level effects of GLP-1RAs on atherosclerosis progression, including inflammation, endothelial function, and plaque stability, is presented in Figure 1.

## 3 GLP-1RA-modulated biomarkers of structural remodeling and plaque instability

Biochemical biomarkers are essential for detecting early vascular dysfunction, monitoring plaque progression, and evaluating therapeutic efficacy. Across reviewed human studies, GLP-1RAs consistently modulate inflammatory cytokines, adhesion molecules, and emerging biomarkers associated with early plaque formation. These biomarker responses provide clinically relevant insight into potential vascular benefits of GLP-1RA therapy. Mechanistically, GLP-1 receptor agonists may exert vascular protective effects by suppressing inflammation, limiting oxidative stress, and modulating key enzymes involved in extracellular matrix remodeling (4).

Matrix metalloproteinases, particularly MMP-9, together with their endogenous inhibitors (TIMPs), play central roles in extracellular matrix turnover and structural vascular remodeling (4). Clinical studies show that GLP-1RA therapy decreases circulating MMP-9 and C-reactive protein (CRP) while increasing TIMP-1 and osteoprotegerin (OPG), indicating concurrent suppression of inflammation and attenuation of proteolytic vascular injury (4). Importantly, the MMP-9/TIMP-1 ratio has emerged as an independent predictor of slower aortic enlargement, supporting the concept that limiting matrix degradation is a key mechanism by which GLP-1RAs may help mitigate vessel wall weakening and plaque destabilization (4).

The reduction in CRP, a recognized marker of aortic stiffness, medial degeneration, and systemic inflammation, further supports the anti-inflammatory profile associated with GLP-1RA therapy. This observation is consistent with evidence demonstrating reductions in high-sensitivity CRP (hs-CRP), highlighting a broader anti-inflammatory and anti-atherosclerotic profile of this drug class (17). Concurrent elevations in OPG, an immunomodulatory member of the TNF receptor superfamily, may represent a compensatory vascular protective response during GLP-1RA treatment (4). Collectively, these molecular and biochemical changes underscore the multifaceted mechanisms by which GLP-1RAs promote vascular stability and counteract atherosclerotic progression, although further longitudinal and plaque-level mechanistic studies are required to confirm direct causal effects.

Beyond these established biomarkers, emerging molecular signatures such as circulating microRNAs (miRs) are increasingly recognized as sensitive indicators of early atherosclerotic changes and may provide additional insight into GLP-1RA-mediated vascular effects.

## 4 Potential emerging biomarkers of early atherosclerotic initiation

Building on these established biomarkers, emerging molecular regulators such as circulating microRNAs (miRs) have gained increasing attention as early indicators of atherosclerotic initiation. Although evidence for direct GLP-1RA regulation of circulating miRs is still emerging, miRs represent an advanced biomarker technology with significant relevance to atherosclerosis biology. Their ability to capture rapid, cell-specific molecular responses positions them as promising candidates for future studies exploring GLP-1RA-mediated molecular signatures.

miRs are small, non-coding RNA molecules that regulate gene expression and are deeply involved in cardiovascular pathophysiology (18, 19). Circulating miRs have gained attention as non-invasive biomarkers capable of detecting early inflammatory and metabolic disturbances preceding structural vascular lesions (18). Unlike conventional biomarkers such as LDL-C or CRP, miRs can reflect dynamic changes in plaque composition, endothelial dysfunction, and vascular inflammation, offering potential for earlier identification of high-risk atherosclerotic lesions (20).

A recent study evaluated 160 circulating plasma miRs in patients with stable coronary artery disease (CAD) and assessed their association with lipid-rich coronary plaques. Among these, miR-133b has been associated with lipid-rich plaques independent of traditional cardiovascular risk factors, suggesting its potential as a biomarker of plaque vulnerability (18). Importantly, this association remained significant after adjustment for traditional cardiovascular risk factors, including age, sex, metabolic status, smoking, and history of cardiovascular disease, supporting its potential role as a biomarker of plaque vulnerability and future myocardial infarction (MI) risk. However, larger prospective cohorts are required to validate the prognostic and clinical utility of miR-133b. Although circulating biomarkers provide insight into systemic inflammatory and remodeling processes, as summarized in Table 1, they may not fully capture localized plaque biology. Variability in assay platforms, sensitivity, and patient heterogeneity further complicates cross-study comparisons. Moreover, biomarker modulation does not necessarily translate into structural plaque modification or clinical event reduction. Integrating circulating biomarkers with imaging-based plaque characterization may provide a more comprehensive assessment of therapeutic vascular effects.

## 5 Imaging and detection technologies in atherosclerosis

### 5.1 High-throughput lipidomics and proteomics for atherosclerosis detection

Advanced molecular profiling technologies, including lipidomics (comprehensive analysis of lipid species) and proteomics (large-scale characterization of circulating proteins), enable simultaneous quantification of numerous plasma components, allowing early detection of atherosclerotic alterations beyond conventional risk factors. High-throughput lipidomic and proteomics can capture complex molecular signatures associated with early vascular injury and disease progression. Techniques such as liquid chromatography–mass spectrometry (LC-MS), direct infusion mass spectrometry, proximity extension assays (Olink), and aptamer-based assays (SomaScan) enable detection of multidimensional biomarker patterns linked to atherosclerosis progression (21).

Key findings include identification of lipid species such as ceramides, sphingolipids, and glycerolipids that strongly correlate with atherosclerosis progression and cardiovascular events (22). Importantly, these lipid species are not only descriptive biomarkers but also form the basis of validated lipid-derived risk scores, such as the ceramide score and CERT2, which have been shown to improve cardiovascular risk prediction beyond traditional lipid measures and to reflect underlying atherosclerotic plaque biology. Lipid-based risk scores, including the ceramide score and CERT2, further improve risk stratification beyond conventional models (21). Supporting this, experimental studies in rabbit models demonstrated that elevated long-chain saturated ceramides in lipoprotein particles are associated with early atherosclerosis severity independent of classical risk factors (23).

Importantly, these platforms provide a scalable approach to evaluate vascular molecular responses to therapies. While direct evidence in the context of GLP-1RA remains limited, these approaches may enable identification of early molecular signatures of treatment response and stratify patients based on underlying plaque biology.

### 5.2 Aptamer-based proteomics for monitoring GLP-1RA-induced molecular changes

Building on high-throughput omics approaches, more targeted proteomic platforms such as aptamer-based technologies (SomaScan®) provide deeper insight into specific protein-level changes associated with GLP-1RA therapy. Aptamers are single-stranded DNA or RNA oligonucleotides that fold into defined three-dimensional structures, enabling high-affinity and high-specificity binding to target proteins in a manner analogous to antibodies (24), thereby allowing scalable profiling of disease-related protein signatures. SomaScan® employs slow off-rate modified aptamers (SOMAmers) as capture reagents to enable highly multiplexed, sensitive quantification of over 1000 proteins across a broad dynamic range in biological fluids and tissues (25).

Recent large-scale proteomic analysis of semaglutide therapy in individuals with overweight or obesity, with or without type 2 diabetes, demonstrated a distinct proteomic signature following treatment (26). Semaglutide reduced proteins involved in vascular inflammation and remodeling, including NT-proBNP, angiopoietin-2 (ANGPT2), thrombospondin-2 (THBS2), macrophage scavenger receptor-1 extracellular domain (MSR1), and tenascin-C (TNC), suggesting vascular benefits beyond weight loss and glycemic control. Conversely, TIMP4 was upregulated, indicating a shift toward reduced extracellular matrix degradation and potential plaque stabilization (26).

These findings support aptamer-based proteomics as a promising translational tool for identifying GLP-1RA-responsive vascular pathways, and monitoring therapeutic molecular responses. However, further studies are required to establish their role in patient stratification and clinical decision-making.

### 5.3 MRI-visible GLP-1RA nanoparticles

While omics approaches provide systemic molecular insights, advanced imaging technologies enable spatial and functional assessment of GLP-1RA effects directly within atherosclerotic plaques. A proteolysis-resistant MRI-visible nanoparticle formulation of GLP-1RA (nano-GLP-1RA) was engineered to target GLP-1R—expressing cells within atherosclerotic lesions (16). Incorporation of gadolinium chelates enabled strong MRI contrast, allowing direct tracking of nanoparticle accumulation.

In ApoE⁻/⁻ mice, nano-GLP-1RA selectively accumulated in plaque-resident inflammatory and de-differentiated smooth muscle cells, consistent with GLP-1R—expressing populations observed in human plaques (16). Imaging confirmed localization to lipid-rich inflamed regions, enabling simultaneous assessment of receptor expression and therapeutic delivery. Targeting is achieved through selective interaction of the GLP-1RA ligand with GLP-1R—expressing cells, including macrophages and smooth muscle cells, resulting in preferential nanoparticle accumulation within plaque-resident inflammatory cell populations (16). Nano-GLP-1RA may reduce local inflammation and promote plaque stabilization by targeting GLP-1R—expressing vascular cells. Theranostic nanoparticle strategies may enable combined targeting and imaging-based monitoring of GLP-1RA vascular effects, supporting the potential for future precision medicine approaches, although clinical translation remains to be established. Nanoparticle-based GLP-1RA delivery provides strong proof-of-concept for plaque-targeted therapy. However, current evidence remains largely preclinical. Human safety, long-term biodistribution, and clinical efficacy in reducing cardiovascular events remain to be established. Translation from controlled animal models to heterogeneous human plaques represents a key future challenge.

Taken together, while imaging and omics technologies offer powerful tools to characterize vascular biology, their integration with GLP-1RA therapy for patient stratification remains an emerging concept that requires further clinical validation.

## 6 Future directions

Despite growing evidence supporting the cardiovascular benefits of GLP-1 receptor agonists, current research remains limited by heterogeneity in study design, variability in drug class and treatment duration, and the absence of standardized plaque-level mechanistic assessments. While most available studies emphasize systemic metabolic improvements, relatively few investigate direct vascular, immune-cell, and plaque-resident effects. Future work should integrate high-resolution plaque imaging and longitudinal CV outcomes to link cellular and molecular effects of GLP-1RAs with plaque stabilization and reduced acute coronary events.

### 6.1 Development of longitudinal, plaque-targeted imaging platforms

Building on emerging MRI-detectable GLP-1RA nanoparticle technologies (16), future studies should assess long-term plaque tracking, intra-plaque drug localization, and dose–response relationships independent of weight loss or glycemic control. Plaque-imaging substudies within GLP-1RA CV trials could reveal therapy-induced changes in plaque composition and stability, directly connecting mechanistic effects to cardiovascular outcomes.

### 6.2 Defining GLP-1RA-specific cellular mechanisms

Key mechanistic questions remain unresolved, including how GLP-1RAs modulate monocyte-to-macrophage differentiation, macrophage polarization (M1→M2), foam-cell formation, and cholesterol efflux within atherosclerotic lesions. Linking these cellular effects to imaging markers of plaque stability may clarify how GLP-1RAs reduce rupture risk and acute coronary events.

### 6.3 Integration with other cardioprotective therapies

Given the expanding use of combined cardiometabolic treatments, it is essential to evaluate interactions between GLP-1RAs and statins, SGLT2 inhibitors, and PCSK9 inhibitors. These agents target complementary pathways in atherosclerosis, including lipid lowering, metabolic regulation, and reduction of cardiovascular risk. Combining biomarker and imaging endpoints could determine whether combination therapy enhances plaque stabilization and cardiovascular protection.

### 6.4 Evaluation in advanced-stage plaque models

Most preclinical studies focus on early lesion development, leaving advanced disease stages underexplored. Future investigations should incorporate models of fibrous-cap thinning, plaque instability, and rupture-prone lesions to determine whether GLP-1RAs protect the full spectrum of atherosclerotic progression.

### 6.5 Testing GLP-1RA on new emerging biomarkers of as initiation

Emerging circulating biomarkers capturing early stages of atherosclerosis, including soluble (pro)renin receptor (sPRR), inflammation-associated miRs such as miR-133b (18, 27), should be studied in high-risk populations. Integration of biomarker profiling with plaque imaging could improve prediction of cardiovascular events and clarify GLP-1RAs preventative plaque initiation or progression.

Collectively, current evidence supports a multi-layered model in which GLP-1RAs exert systemic immunometabolic benefits alongside potential direct vascular effects. However, the relative contribution of metabolic vs. plaque-specific mechanisms remains incompletely defined. Addressing this gap will require integrative clinical studies combining omics-based biomarker profiling, high-resolution plaque imaging, and long-term cardiovascular outcome assessment.

## 7 Conclusion

GLP-1 receptor agonists exhibit a multifaceted capacity to modulate the biological processes driving atherosclerosis. Beyond their metabolic actions, GLP-1RAs directly influence vascular tissues by reducing inflammation, oxidative stress, and endothelial activation, each of which contributes to plaque initiation and progression. These layered mechanisms involve both systemic immunometabolic improvement and local receptor-mediated effects at the plaque level, particularly within smooth muscle—derived and immune cells.

Advances in imaging, transcriptomics, and circulating biomarker technologies now offer new opportunities to characterize vascular effects *in vivo*, including reductions in vascular inflammation and oxidative stress, improvements in endothelial function, and modulation of plaque composition and stability. Such tools may ultimately enable patient stratification based on plaque biology rather than traditional risk markers alone. Among emerging evidence, nanoparticle-delivered GLP-1RA formulations provide compelling preclinical evidence of plaque-targeted action. These studies demonstrate that localized GLP-1R activation enhances cholesterol efflux, reduces plaque lipid burden, and suppresses inflammatory gene expression even at doses too low to alter systemic metabolic parameters. This supports a model in which GLP-1RAs may exert direct plaque-modifying effects, although definitive human plaque-level confirmation remains limited.

Importantly, integrating these imaging and omics-based approaches with GLP-1RA therapy may allow identification of patients with inflammation-driven or lipid-rich plaque phenotypes who are more likely to benefit from treatment. In addition, longitudinal monitoring of circulating biomarkers and proteomic signatures could provide a means to assess therapeutic response at the molecular level, enabling more precise and personalized cardiovascular risk stratification.

Altogether, these insights position GLP-1RAs as promising modulators of atherosclerotic disease and highlight the need for targeted mechanistic and clinical research to fully define their vascular impact. Future integrative studies combining multi-omics profiling, high-resolution plaque imaging, and long-term cardiovascular outcome assessment will be essential to determine whether GLP-1RAs can directly modify plaque biology and improve clinical outcomes.
